# Association of Molecular Senescence Markers in Late-Life Depression With Clinical Characteristics and Treatment Outcome

**DOI:** 10.1001/jamanetworkopen.2022.19678

**Published:** 2022-06-30

**Authors:** Breno S. Diniz, Benoit H. Mulsant, Charles F. Reynolds, Daniel M. Blumberger, Jordan F. Karp, Meryl A. Butters, Ana Paula Mendes-Silva, Erica L. Vieira, George Tseng, Eric J. Lenze

**Affiliations:** 1 UConn Center on Aging, University of Connecticut, Farmington; 2 Department of Psychiatry, University of Connecticut School of Medicine, Farmington; 3 Centre for Addiction and Mental Health, Toronto, Ontario, Canada; 4 Department of Psychiatry, Temerty Faculty of Medicine, University of Toronto, Toronto, Ontario, Canada; 5 Department of Psychiatry, University of Pittsburgh School of Medicine, Pittsburgh, Pennsylvania; 6 Department of Psychiatry, The University of Arizona College of Medicine, Tucson; 7 Department of Biostatistics, University of Pittsburgh School of Public Health, Pittsburgh, Pennsylvania; 8 Department of Psychiatry, Washington University in St Louis, St Louis, Missouri

## Abstract

**Importance:**

Many older adults with depression do not experience remission with antidepressant treatment, and markers of cellular senescence in late-life depression (LLD) are associated with greater severity of depression, greater executive dysfunction, and higher medical illness burden. Since these clinical characteristics are associated with remission in LLD, molecular and cellular senescence abnormalities could be a possible biological mechanism underlying poor treatment response in this population.

**Objective:**

To examine whether the senescence-associated secretory phenotype (SASP) index was associated with the likelihood of remission from a depressive episode in older adults.

**Design, Setting, and Participants:**

A nonrandomized, open-label clinical trial was conducted between August 2009 and August 2014 in Pittsburgh, Pennsylvania; St Louis, Missouri; and Toronto, Ontario, Canada, with older adults in a current major depressive episode according to the *Diagnostic and Statistical Manual of Mental Disorders* (Fourth Edition, Text Revision) diagnostic criteria. Data from biomarker analyses were reported according to the clinical trial archived plasma samples run in March 2021. Data were analyzed from June to November 2021.

**Exposure:**

Venlafaxine extended release (dose ranging from 37.5 mg to 300 mg daily) for up to 12 weeks.

**Main Outcomes and Measures:**

The association between a composite biomarker-based index (SASP index) and treatment remission in older adults with major depression was measured using clinical data and blood samples.

**Results:**

There were 416 participants with a mean (SD) age of 60.02 (7.13) years; 64% (265 participants) were self-reported female, and the mean (SD) Montgomery-Asberg Depression Rating Scale score was 26.6 (5.7). Higher SASP index scores were independently associated with higher rates of nonremission, with an increase of 1 unit in the SASP index score increasing the odds of nonremission by 19% (adjusted odds ratio, 1.19; 95% CI, 1.05-1.35; *P* = .006). In contrast, no individual SASP factors were associated with remission in LLD.

**Conclusions and Relevance:**

Using clinical data and blood samples from a nonrandomized clinical trial, the results of this study suggest that molecular and cellular senescence, as measured with the SASP index, is associated with worse treatment outcomes in LLD. Combining this index score reflecting interrelated biological processes with other molecular, clinical, and neuroimaging markers may be useful in evaluating antidepressant treatment outcomes. These findings inform a path forward for geroscience-guided interventions targeting senescence to improve remission rates in LLD.

**Trial Registration:**

ClinicalTrials.gov Identifier: NCT00892047

## Key Points

**Question:**

Are markers associated with the senescence-associated secretory phenotype (SASP) associated with treatment remission in older adults with major depressive episode?

**Findings:**

In this nonrandomized clinical trial of 416 participants, higher SASP index, a composite score of 22 biomarkers associated with the SASP, was significantly associated with lower likelihood of remission to antidepressant treatment.

**Meaning:**

These findings suggest that molecular and cellular senescence abnormalities could be a putative mechanism associated with worse treatment outcomes in older adults with a major depressive episode.

## Teaser

This nonrandomized clinical trial evaluates the association between molecular senescence markers and treatment remission in older adults with depression.

## Introduction

Achieving response and remission in older adults with major depressive disorder (late-life depression [LLD]) is a major clinical challenge. Treatment response and remission rates in LLD are approximately 50% after first-line antidepressant treatment.^1^ Nonremission leads to persistent depressive symptoms, a source of depleted psychological well-being, increased disability, accelerated cognitive decline, and premature mortality in older adults.^2^ Therefore, identifying mechanisms and factors associated with treatment outcomes is key for improving therapeutics and for a priori identification of individuals for whom antidepressant treatment would be more effective. A recent meta-analysis^3^ identified higher baseline depression severity, comorbid anxiety, and executive dysfunction as the most important clinical variables associated with poor treatment response in LLD. Other studies^4,5,6,7^ have also identified cardiovascular risk factors (eg, metabolic syndrome), cerebrovascular disease, brain structural changes, physical comorbidity, and frailty as variables associated with poor treatment response. Some of these variables are common in depression across the lifespan (eg, comorbid anxiety and depression severity), but others are geriatric specific (eg, cerebrovascular disease burden and executive dysfunction), suggesting that remission with treatment in LLD may have specific mechanisms that are associated with age-related brain and systemic aging processes.

From a geroscience perspective, identifying and targeting biological processes associated with biological aging can ideally prevent, or at a minimum delay, the onset and progression of multiple chronic diseases and adverse age-related health outcomes that are typically observed in older adults.^8,9^ Previous works have suggested the involvement of biological aging and cellular senescence abnormalities in the biological mechanisms of LLD. For example, abnormalities in proteostasis control, heightened proinflammatory status, mitochondrial dysfunction, insulin resistance, and metabolic dysregulation have been described in LLD. They are associated with cognitive impairment, physical comorbidity burden, and higher mortality risk.^10,11,12,13,14,15^ Cellular senescence and changes in its cellular secretome (ie, the senescence-associated secretory phenotype or SASP) are other important hallmarks of biological aging.^16^ The SASP factors comprise several signaling proteins associated with inflammatory control, tissue remodeling, cell growth, cell cycle control, and metabolic regulation. They act in concert in different tissues and cell types, propagating prosenescence signals, leading to cellular senescence changes in neighboring cells and distant tissues.^17,18^ Reflecting these multiple interrelated biological pathways and functions, examining them as a biomarker-composite index may be more robust than examining each individual factor separately for examining disease outcomes, much as polygenic risk scores are more predictive than a single source of genetic variation.^19^ For example, recent studies have shown that a SASP index, including 22 SASP factors, is substantially elevated in LLD and associated with the severity of depressive symptoms, executive dysfunction, the burden of physical comorbidity, and markers of worse brain health.^20,21,22,23^

Biological mechanisms of aging itself may drive LLD treatment outcomes. A genome-wide association study,^24^ including 184 individuals treated with venlafaxine extended release (XR), has identified 8 loci associated with treatment remission in LLD. The most common biological pathways associated with these genes were proteostasis regulation (eg, ubiquitin-proteasome system) and vascular health. Another recent study,^25^ including 64 older adults with LLD, investigated the transcriptomic profile associated with remission with escitalopram and memantine treatment and showed that both medications strongly modulated inflammatory responses. Other biological pathways associated with remission were cellular proliferation, apoptosis (escitalopram only), cellular clearance, metabolism, and cytoskeletal dynamics (escitalopram and memantine). These studies point to processes implicated in accelerated biological aging (ie, immunoinflammatory response, cellular proliferation and apoptosis, and metabolic control) as mechanisms and variables associated with treatment response and remission in LLD.^16^

Thus, we undertook an analysis to evaluate whether SASP factors, systemic indicators of abnormal cellular senescence, were associated with treatment remission in a large sample of older adults with LLD. Our primary hypothesis was that a higher composite score of SASP factors (the SASP index) would be associated with treatment resistance (ie, nonremission to venlafaxine XR treatment). We also evaluated the association between the SASP index and demographic and clinical characteristics.

## Methods

In this nonrandomized controlled trial, we analyzed blood samples and clinical data from participants in the open-label phase of the Incomplete Response in Late-Life Depression: Getting to Remission (IRLGREY) Study, a 3-site trial in which treatment remission was assessed prospectively.^26^ This study reports secondary data analyses focusing on the association between blood-based biomarkers and treatment remission according to data available from the IRLGREY study. The prespecified primary and secondary outcomes have been previously published.^26,27^ Participants provided written informed consent, and ethics approval was obtained from the institutional review boards at the Centre for Addiction and Mental Health in Toronto, the University of Pittsburgh, and Washington University in St Louis. This study follows the Transparent Reporting of Evaluations With Nonrandomized Designs (TREND) reporting guideline. The trial protocol is available in Supplement 1.

### Study Sample

The IRLGREY study, conducted between August 2009 and August 2014, has been described in detail previously.^26^ Participants were aged 60 years or older and had current nonpsychotic major depressive disorder (MDD) according to the *Diagnostic and Statistical Manual of Mental Disorders* (Fourth Edition, Text Revision) (*DSM-IV-TR*) diagnostic criteria^28^ with a score of 15 or higher on the Montgomery-Asberg Depression Rating Scale (MADRS).^29^ The diagnosis of MDD and the presence of a major depressive episode were confirmed with the Structured Clinical Interview for *DSM-IV-TR* Axis I Disorders (SCID-IV-TR), Research Version, Patient Edition.^30^ Exclusions were lifetime diagnosis of bipolar disorder, schizophrenia, schizoaffective disorder, other psychotic disorders, or current psychotic symptoms; clinical history of dementia; alcohol or substance use disorder in the past 6 months; imminent suicide risk; unstable physical illness; or contraindication to venlafaxine XR.

### Intervention

Participants were treated for up to 12 weeks openly with venlafaxine XR. Participants started at 37.5 mg per day and flexibly titrated as needed up to 300 mg per day following a standardized protocol as published previously.^26^

### Outcome Measure and Additional Demographic and Clinical Variables

Depression symptom severity was measured by the MADRS score assessed at study baseline, week 1, week 2, and every 2 weeks thereafter for 12 weeks, with remission defined as MADRS scores of 10 or below for 2 consecutive assessments at phase end.

Demographic (age, self-reported sex, self-reported race [Black, White], years of education) and anthropometric (weight, height) data were obtained in the baseline assessment. Race was assessed in this study because it is a social variable that can influence access to treatment and the antidepressant remission rates. Body mass index (BMI) was calculated as weight in kilograms divided by height in meters squared. Blood pressure was measured while sitting after at least 5 minutes of resting. Systolic and diastolic blood pressure values are the mean of 2 measurements taken 5 minutes apart. Physical comorbidity burden was assessed with the Cumulative Illness Rating Scale–Geriatrics.^31^

Information about the participant’s history, including the length of the current depressive episode, age of onset of the first depressive episode, depressive episode recurrence, and presence of comorbid anxiety disorder, was obtained during the SCID-IV-TR interview. In addition, the MDD was classified as early-onset depression (EOD) or late-onset depression (LOD) depending on whether the first depressive episode happened before or after the age of 60 years.

A neuropsychological test battery was administered before starting venlafaxine XR. Executive function was evaluated using 2 tests from the Delis-Kaplan Executive Function System—the Color-Word Interference task (measuring response inhibition) and the Trail Making Test (measuring set-shifting and cognitive flexibility).^32^ The Color-Word Interference condition 3, called inhibition, assesses the ability to inhibit an automatic response (ie, reading words); instead, participants must produce a response that requires more effort (ie, naming the colors of words). The Trail Making Test condition 4 (also known as the Number-Letter Switching condition) requires participants to switch back and forth between connecting numbers and letters (ie, 1-A, 2-B, to 16-P). Condition 5 is a motor speed condition in which participants trace over a dotted line connecting circles on the page as quickly as possible to gauge their motor drawing speed. Comparing performance on condition 4 (which assesses cognitive flexibility) with performance on condition 5 (which assesses motor speed) removes the motor speed element from the test score to ascertain cognitive flexibility^33^; thus, to evaluate set-shifting performance, we used the Delis-Kaplan Executive Function System normed scaled score (with a mean [SD] score of 10 [3]) for the difference in speeds between condition 4 and condition 5.^34^ All tests were scored according to published age-adjusted norms for each test,^28,29,30^ and additional details about the neuropsychological testing and scoring can be found elsewhere.^27^

### SASP Factors

Blood was collected by venipuncture with EDTA tubes after overnight fasting and processed immediately after collection. Plasma was obtained from the blood by centrifugation at 3000 *g* for 10 minutes at 4 °C. Plasma was separated, aliquoted, and stored in a −80 °C freezer until the laboratory analysis.

The SASP factors were analyzed by a customized multiplex assay (R&D System) using the Luminex 100/200 platform (Luminex). All the experiments were performed according to the manufacturer's instructions. All the biomarkers were analyzed using the same assay batch, and the coefficient of variation was less than 10% for all analytes. All samples were analyzed on the same day to reduce variability across laboratory experiments.

The SASP factors included in the SASP index are the *IGFBP6*, *IGFBP2*, *CCL4*, interleukin-1β, granulocyte-macrophage-colony-stimulating factor, placental growth factor, angiogenin, migration inhibitory factor–1, macrophage inflammatory protein (MIP)–1α, chemokine growth-regulated protein α, interleukin-6, human monocyte chemoattractant protein–4, glycoprotein 130, intercellular adhesion molecule–1, monocyte chemoattractant protein-1, interleukin-8, MIP-3α, osteoprotegerin, metallopeptidase inhibitor–1, urokinase-type plasminogen activator receptor, tumor necrosis factor (TNF) receptor–I, and TNF receptor–II. We selected the candidate SASP proteins included in our analyses according to previous preclinical studies focused on the changes in the secretome pattern of senescent cells^35^ and our previous publications.^20,22^ The raw data were log_2_ transformed and standardized to the *z* score. We calculated the SASP index score for each participant according to the following regression formula: SASP index = β_1_*x*_1_ + … + β_22_*x*_22_, where β is the individual weight and *x* is the standardized value of each biomarker included in the SASP index. The weight for each factor was derived from a previous publication^20^ from our group using an independent and clinically heterogeneous sample of older adults with and without a history of MDD (eTable 1 in Supplement 2). The SASP index mean was centered at 0, with an SD of 1 in the whole sample.

### Statistical Analysis

First, we conducted a descriptive analysis of all the continuous variables and the visual inspection of P-P plots to evaluate whether they followed a normal distribution. The variables that did not show a normal distribution were log-transformed. Then, we performed 2-sided *t* tests to assess the association between demographic variables (eg, sex) or characteristics of the MDD (eg, LOD vs EOD) and the SASP index scores. We also performed Pearson correlation analyses to evaluate the association between SASP index scores, baseline demographics, clinical data, and executive function performance data.

We evaluated whether treatment outcome (nonremission vs remission) was associated with differences in SASP index score or other baseline demographic, clinical, and executive function performance data using *t* tests. Analyses of covariance were done to evaluate the relevance of potential covariates on the association between treatment outcome and the SASP index score. Finally, we performed a logistic regression analysis to assess potential variables associated with treatment outcome to venlafaxine XR (treatment resistance as the reference group). First, we evaluated whether the SASP index scores baseline demographic, clinical, and executive function performance data were associated with treatment resistance in unadjusted models (a total of 12 analyses were run in the unadjusted logistic regression analyses). Then, variables that were significantly associated with treatment resistance in the unadjusted model (ie, variables with *P* < .05) were entered simultaneously in the model (adjusted model). We did not test the interaction between the SASP index and covariates in the logistic regression models. We used the Hosmer-Lemeshow test to evaluate the goodness-of-fit of the model. We also did the same set of analyses with each SASP factor. All analyses were performed using Stata statistical software version 17 for Windows (StataCorp). Data were analyzed from June to November 2021.

## Results

### SASP Index and Baseline Characteristics

Participants totaled 416 and were included in this analysis if they had complete data from baseline and at least 1 follow-up visit, plus baseline biomarker data. Participants’ mean (SD) age was 60.0 (7.1) years, 64% (265 participants) were self-reported female, and the mean (SD) MADRS score was 26.6 (5.7). Table 1 shows the demographic and clinical characteristics of the sample, broken down by their remission status.

**Table 1.** Sample Characteristics According to Treatment Outcome After Treatment With Venlafaxine Extended Release

Characteristic	Patients, No. (%), by treatment outcome		Statistics	*P* value
	Nonremission (n = 240)	Remission (n = 176)		
Self-reported sex				
Female	139 (58)	126 (72)	χ^2^_1_ = 7.74	.005
Male	101 (42)	50 (28)		
Age, mean (SD), y	68.6 (6.9)	69.7 (7.4)	*t*_414_ = 1.54	.12
Education, mean (SD), y	14.2 (2.8)	14.7 (2.8)	*t*_414_ = 1.65	.10
Self-reported race				
Black	29 (12)	17 (10)	χ^2^_1_ = 0.35	.55
White	211 (88)	159 (90)		
Montgomery-Asberg Depression Rating Scale score, mean (SD)	27.7 (5.7)	25.1 (5.4)	*t*_414_ = 4.72	<.001
Duration of current major depressive episode, mean (SD), d	342 (673)	215 (508.8)	*t*_414_ = 2.10	.04
Comorbid anxiety diagnosis				
No	139 (58)	104 (59)	χ^2^_1_ = 0.24	.87
Yes	101 (42)	72 (41)		
Age of onset of major depressive disorder				
Late onset depression	68 (28)	51 (29)	χ^2^_1_ = 2.91	.09
Early onset depression	172 (72)	126 (71)		
CIRS-G, Cumulative Illness Rating Scale-–Geriatric total score, mean (SD)	10.1 (4.6)	9.6 (4.2)	*t*_414_ = 1.18	.23
Body mass index, mean (SD)^a^	29.8 (7.3)	29.6 (6.0)	*t*_414_ = 0.25	.73
Blood pressure, mean (SD), mm Hg				
Systolic	133.4 (19.4)	130.3 (18.7)	*t*_414_ = 0.92	.10
Diastolic	77.5 (11.6)	74.7 (11.5)	*t*_414_ = 2.40	.02
Set shifting score, mean (SD)	8.5 (4.1)	8.9 (4.1)	*t*_414_ = 0.93	.35
Response inhibition score, mean (SD)	10.4 (3.1)	10.2 (2.9)	*t*_414_ = 0.65	.52
Response inhibition-shifting score, mean (SD)	9.9 (3.4)	10.2 (3.7)	*t*_414_ = 1.01	.31
Senescence-associated secretory phenotype index score, mean (SD)	0.25 (0.10)	−0.19 (0.14)	*t*_414_ = 2.58	.01

^a^ Body mass index is calculated as weight in kilograms divided by height in meters squared.

**Figure 1.**
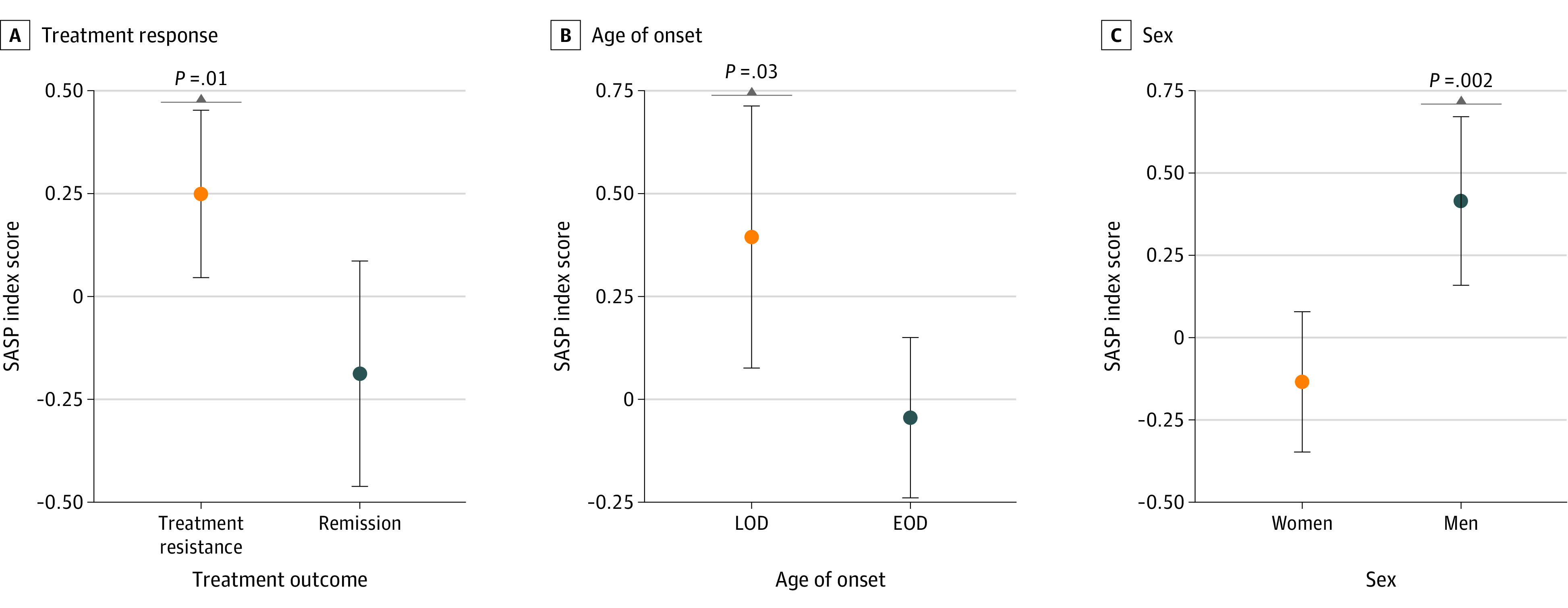
Senescence-Associated Secretory Phenotype (SASP) Index Scores According to Treatment Response, Age of Onset of the Depressive Disorder, and Sex Dots indicate mean and whiskers indicate 95% CI. EOD indicates early-onset depression; LOD, late-onset depression.

**Figure 2.**
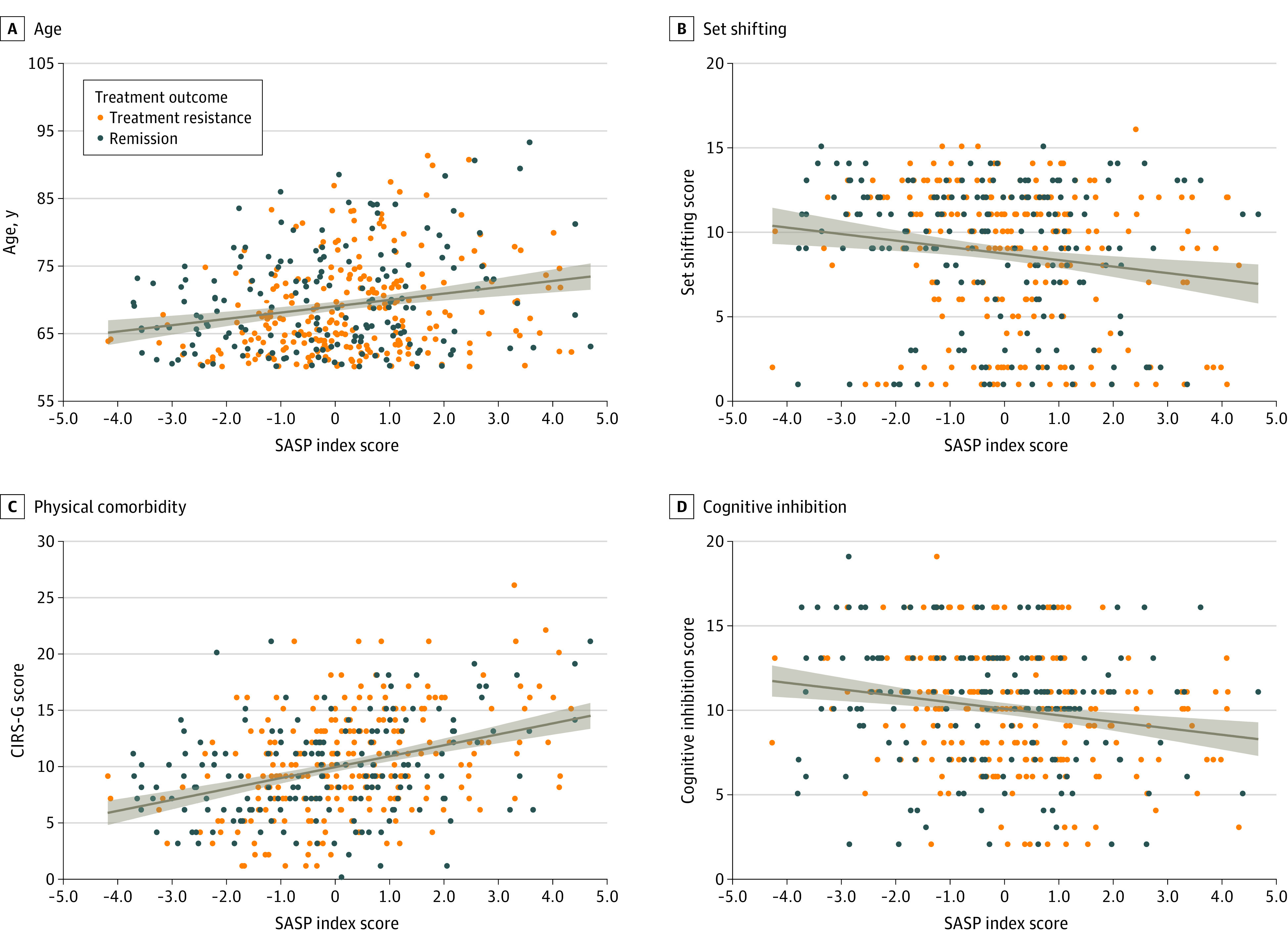
Scatterplots of the Association Between Senescence-Associated Secretory Phenotype (SASP) Index Scores, Age, Medical Comorbidity Burden and Executive Function Performance Dots indicate each individual included in the analysis; shaded area indicates 95% CI for the linear trend; line indicates linear trend. CIRS-G indicates Cumulative Illness Rating Scale–Geriatrics.

The 103 participants with LOD had significantly higher SASP index scores than the 313 participants with EOD (mean [SD], 0.39 [0.16] vs −0.05 [0.10]; *t*_414_ = 2.25; *P* = .02). Self-reported male sex was significantly associated with higher SASP index scores (mean [SD], 0.41 [0.12] for male vs −0.13 [0.11] for female; *t*_414_ = 3.16; *P* = .002). (Figure 1). In the full sample, higher SASP index scores were associated with older age (*r* = 0.22; *P* < .001), higher physical comorbidity burden (*r* = 0.37; *P* < .001), higher BMI (*r* = 0.23; *P* < .001), higher systolic blood pressure (*r* = 0.11; *P* = .02), and worse performance on executive function tests (set shifting score *r* = −0.16; *P* = .001; inhibition score, *r* = −0.19; *P* < .001; inhibition-switching score, *r* = −0.19; *P* < .001) (Figure 2). The SASP index scores were not significantly associated with MADRS scores (*r* = −0.03; *P* = .53), duration of the current depressive episode (*r* = 0.07; *P* = .14), educational level (*r* = −0.09; *P* = .07), or diastolic blood pressure measures (*r* = −0.02; *P* = .60).

### SASP Index and Treatment Outcomes With Venlafaxine XR

With open-label treatment with venlafaxine XR, 176 participants achieved remission (remission group) and 240 did not (nonremission group). Participants in the nonremission group were more frequently male, had higher pretreatment MADRS scores, longer current depressive episode, higher diastolic blood pressure, and higher SASP index scores than those in the remission group (Table 1). The association between the SASP index scores and nonremission remained significant (*F*_5,407_ = 5.37; *P* = .02) after controlling for the effect of sex (*F*_5,407_ = 8.36; *P* = .004), baseline depressive symptoms (*F*_5,407_ = 0.05; *P* = .80), length of the current depressive episode (*F*_5,407_ = 1.09; *P* = .29), and diastolic blood pressure levels (*F*_5,407_ = 1.73; *P* = .19). (Figure 1). Since sex and age of onset were associated with SASP index, we also tested whether they were moderators of the association between treatment outcome and SASP index. Neither sex (*F*_3,412_ = 2.81; *P* = .09) nor age of onset of MDD (*F*_3,412_ = 0.04; *P* = .84) significantly moderated the association between treatment outcomes and the SASP index.

Since the SASP index includes 22 independent biomarkers, we also explored the association between each biomarker and remission status. We found that the MIP-3α and TNFRII were significantly higher in those with nonremission (eTable 1 in Supplement 2). However, this association did not remain significant after controlling for the covariates (sex, severity of baseline depressive symptoms, length of current depressive episode, and diastolic blood pressure) (eTable 1 in Supplement 2).

In the logistic regression analysis, a higher SASP index score was associated with a higher risk of nonremission, with an increase of 1 unit of the SASP index scores, increasing the risk of nonremission by 16% (odds ratio [OR], 1.16; 95% CI_,_ 1.06-1.30; Wald = 6.47; *P* = .01). In the adjusted model, including sex, MADRS scores, and cognitive inhibition and set-shifting scores, the SASP index scores remained independently associated with a 19% higher risk of nonremission of the major depressive episode (OR, 1.19; 95% CI, 1.05-1.35l; Wald = 7.48; *P* = .006) (Table 2). We also evaluated the association between each 22 independent SASP biomarkers and treatment outcomes. Again, the only biomarker that was associated with nonremission was the MIP-3α (OR, 1.11; 95% CI, 1.00-1.23; Wald = 4.08; *P* = .045). However, this association did not remain significant in the adjusted model (OR, 1.09; 95% CI, 0.98-1.22; Wald = 2.46; *P* = .12) (eTable 2 in Supplement 2).

**Table 2.** Logistic Regression to Determine Factors Associated With Treatment Outcome

Variable	Unadjusted			Adjusted^a^		
	OR (95% CI)	Wald	*P* value	OR (95% CI)	Wald	*P* value
Senescence-associated secretory phenotype index score	1.16 (1.06-1.30)	6.47	.01	1.19 (1.05-1.35)	7.48	.005
Self-reported sex	2.060 (1.26-3.37)	8.243	.004	1.86 (1.23-3.11)	8.00	.005
MADRS score	0.908 (0.87-0.95)	19.99	<.001	1.10 (1.06-1.14)	23.48	<.001
Duration of MDE	1.000 (0.99-1.00)	3.557	.06	NA	NA	NA
Age	1.007 (0.97-1.04)	.141	.71	NA	NA	NA
Self-reported race	1.099 (0.51-2.37)	.058	.81	NA	NA	NA
Years of education	1.077 (0.99-1.17)	2.915	.09	NA	NA	NA
CIRS-G	.956 (0.90-1.01)	2.501	.11	NA	NA	NA
Set shifting	1.117 (1.00-1.24)	4.246	.04	1.06 (1.00-1.13)	3.87	.05
Response inhibition	.867 (0.78-0.96)	7.702	.006	1.10 (1.01-1.19)	5.21	.02
Response inhibition-shifting	1.077 (0.99-1.18)	2.741	.01	NA	NA	NA
Comorbid anxiety diagnosis	.941 (0.59-1.49)	.066	.79	NA	NA	NA
Blood pressure						
Systolic	1.000 (0.99-1.01)	.001	.98	NA	NA	NA
Diastolic	.983 (0.96-1.01)	1.763	.18	NA	NA	NA
BMI^b^	1.014 (0.98-1.05)	.562	.453	NA	NA	NA

Abbreviations: BMI, body mass index; CIRS-G, Cumulative Illness Rating Scale-Geriatric; MADRS, Montgomery-Asberg Depression Rating Scale; MDE, current major depressive episode; NA, not applicable; OR, odds ratio.

^a^ Adjusted by self-reported male sex, MADRS scores, and scores on cognitive inhibition and set-shifting tasks. OR greater than 1.0 indicates a higher risk of nonremission status.

^b^ Body mass index is calculated as weight in kilograms divided by height in meters squared.

## Discussion

In this nonrandomized controlled trial, we explored whether circulating molecular markers of cellular senescence (SASP factors) were prospectively associated with nonremission to venlafaxine treatment in a large sample of older adults with MDD. A higher SASP index composite score was a significant and independent variable associated with nonremission in this population. By contrast, none of the individual SASP biomarkers was independently associated with remission status. We also showed that higher SASP index scores were associated with specific characteristics of the depressive episode as a geriatric syndrome, namely, later age of onset, a higher burden of medical comorbidity, and executive dysfunction. This is important because it demonstrates that the key interrelated biological processes of aging called senescence may underlie many cases of LLD, particularly in the context of poor treatment response. This finding is concordant with research on late-life neuropsychiatric disorders and could lead to geriatric-specific solutions to improve treatment outcomes in mental disorders by interrogating and intervening at the level of cellular aging.^36,37^

Inflammatory markers constitute a significant component of the SASP index,^38^ and several studies have addressed whether inflammatory markers are associated with antidepressant treatment outcomes, with mixed results. For example, in an early study of younger adults with MDD treated with escitalopram for 12 weeks, higher serum TNF-α was associated with a worse treatment response.^39^ However, in 2 other studies, plasma levels of TNF-α, TNF-α receptors, or other inflammatory biomarkers (eg, C-reactive protein, interleukin-6) did not project response or remission to treatment with sertraline, transcranial direct current stimulation, or omega-3 fatty acid.^40,41^ In a study of 52 young participants with MDD treated with venlafaxine, none of 7 inflammatory markers were individually associated with treatment response.^42^ However, recent meta-analyses^43,44^ suggested that higher levels of interleukin-8 and C-reactive protein are associated with worse treatment response in MDD, although these associations had small effect sizes. All these studies evaluated whether individual biomarkers were associated with outcome; none assessed whether a composite index was associated with antidepressant treatment outcome, and they focused mostly on a young and middle-aged adult population.

Like these other studies, none of our individual inflammatory (or other) biomarkers was associated with remission with antidepressant treatment. By contrast, when all these biomarkers were incorporated into a single composite index (SASP index) associated with different pathways affected by pathological aging processes (eg, inflammation, metabolic control, cell growth, and tissue remodeling), the SASP index showed a robust association with remission in LLD. Our findings, thus, provide evidence that moving beyond the 1 disease, 1 molecule paradigm by incorporating multiple biomarkers reflecting different biological pathways into a composite index can be more powerful in informing the biological mechanisms associated with treatment outcomes for psychiatric disorders. Other examples in psychiatry include the development of polygenic risk scores to investigate the mechanisms and biological variables associated with treatment response in major depression.^45,46,47^

Previous works have explored the association between telomere length, a robust marker of cellular senescence, and MDD across the lifespan. Overall, there is evidence of shorter leukocyte telomere length in patients with MDD, with mild to moderate effect size (Cohen *d* = −0.205).^48^ Also, a recent study^49^ reported a substantial association between the severity of the depressive episode in LLD and shorter leukocyte telomere length. A few small clinical trials^50,51,52^ evaluated whether leukocyte telomere length was associated with treatment response or remission in MDD, with mixed results. None of these studies included older adults with LLD, a population more vulnerable to age-related biological abnormalities.^53^ Our previous study^18^ evaluated the association of molecular and cellular senescence with the outcome of protocolized antidepressant treatment in a large sample of older patients with MDD. We focused on well-established circulating SASP factors.^15,16,17^ Our finding that a higher SASP index score, reflecting more intense molecular and cellular senescence abnormalities, is associated with treatment outcome supports the hypothesis that enhanced age-related biological changes render older individuals with LLD less prone to achieve remission of the depressive episode.

These previous results and our congruent findings suggest that molecular and cellular senescence abnormalities may be a common mechanism negatively associated with treatment outcomes indirectly and directly. Cellular senescence and the SASP are viewed as responses to different stressors that lead to cell dysfunction (eg, DNA damage and loss of proteostasis).^54,55^ The accumulation of senescent cells leads to a gradual loss of the capacity of tissues to optimally compensate homeostatic function against deleterious stimuli and recover normal function even after removing the noxious stimuli.^56,57^ The accumulation of senescent cells in different tissues and SASP factors systemically can lead to the incidence of various age-related diseases and worse medical outcomes.^17,58^ In the context of our study, we can speculate that a higher SASP index is a general indicator of systemic and brain dysfunction that leads to a worse response to antidepressants. Moreover, these poor outcomes observed in LLD can also reflect a broader process of unhealthy aging that can be identified by an elevated SASP index. Interventions and lifestyle modifications, including increased physical activity, improved dietary patterns, and good management of chronic medical conditions, contribute to improved general health, possibly by regulating different age-related biological processes and slowing of molecular and cellular senescence.^59,60,61^ Importantly, these lifestyle and behavioral factors are protective against the development of depressive symptoms and can be used as adjunctive treatment options to improve treatment response in older adults with major depression,^62,63,64^ but also can be useful for the prevention of other age-related outcomes commonly observed in this population. Finally, geroscience-guided interventions, such as using senolytic drugs that specifically clear senescence cells aiming to reduce or mitigate cellular senescence and the harmful effects of SASP factors,^65^ could be used to improve antidepressant treatment outcomes in LLD. Such strategies have recently showed promising results to mitigate anxiety and cognitive impairment after chronic stress in different animal models.^37,66^

Several variables were significantly associated with a higher SASP index among the LLD participants. Self-reported sex was associated with the SASP index scores, with male participants having higher SASP index scores than female participants. A meta-analysis showed that biological sex was not significantly associated with leukocyte telomere length and MDD.^67^ Our study offers additional evidence that biological sex can be significantly associated with biological hallmarks of aging in LLD, with male individuals showing a higher SASP index score. This finding could explain the higher risk of poorer outcomes, such as higher mortality, in older male individuals with MDD compared with female individuals.^68^ Several lines of evidence suggest that patients with late-onset depression and early-onset depression have distinct biological mechanisms^69^ and long-term, age-related outcomes (eg, more rapid cognitive decline in LOD vs EOD).^70^ In our study, participants with LOD had higher SASP than EOD, again providing additional evidence for potential distinct mechanisms between these 2 subgroups of LLD.

### Strengths and Limitations

Our study has some strengths (eg, a large sample and the use of protocolized antidepressant treatment to prospectively characterize outcome) and limitations. First and foremost, all participants were treated with venlafaxine XR, and we cannot distinguish whether the association between higher SASP index scores and nonremission is specific to venlafaxine or reflects a more general effect of molecular and cellular senescence on treatment remission in LLD. We did not have a specific measure of vascular risk scores in this sample, but a more systemic measure of medical burden (the Cumulative Illness Rating Scale–Geriatrics), and we may not have more detailed information about the association between the SASP index and vascular risk factors. Also, our sample mainly included White participants, and our results may not be generalizable to other racial or ethnic groups. Another potential limitation was the inclusion of participants who have a history of alcohol use disorder as long as it was in remission for at least 6 months. This duration may not be sufficient to rule out the impact of alcohol use disorder on senescence markers. However, we have previously reported on the absence of association between previous or current alcohol use and SASP index.^21^ Thus, it is unlikely that the inclusion of participants with a history of alcohol use disorder influenced the current results.

## Conclusions

In conclusion, our findings suggest that molecular and cellular senescence, as measured with the SASP index, is significantly associated with worse treatment outcomes in LLD. Our study also demonstrates that a composite index, integrating biomarkers reflecting distinct but interrelated biological processes, is superior to any single biomarkers in the association with treatment remission in LLD. Our findings can inform a path forward for geroscience-guided interventions targeting senescence to improve remission rates in LLD.

## Notes

Supplement 1.
Trial Protocol

Supplement 2. **eTable 1.** Comparison Between Individual SASP Factors Between Nonremission vs Remission Groups **eTable 2.** Logistic Regression of Each Individual SASP Factor to Predict Treatment Outcome

## References

[1] Lenze EJ, Sheffrin M, Driscoll HC, . Incomplete response in late-life depression: getting to remission. Dialogues Clin Neurosci. 2008;10(4):419-430. doi:10.31887/DCNS.2008.10.4/jlenze 19170399 PMC3181898

[2] Taylor WD. Clinical practice: depression in the elderly. N Engl J Med. 2014;371(13):1228-1236. doi:10.1056/NEJMcp1402180 25251617

[3] Tunvirachaisakul C, Gould RL, Coulson MC, . Predictors of treatment outcome in depression in later life: a systematic review and meta-analysis. J Affect Disord. 2018;227:164-182. doi:10.1016/j.jad.2017.10.008 29100149

[4] Brown PJ, Ciarleglio A, Roose SP, . Frailty and depression in late life: a high-risk comorbidity with distinctive clinical presentation and poor antidepressant response. J Gerontol A Biol Sci Med Sci. 2022;77(5):1055-1062. doi:10.1093/gerona/glab338 34758065 PMC9071391

[5] Emam H, Steffens DC, Pearlson GD, Wang L. Increased ventromedial prefrontal cortex activity and connectivity predict poor sertraline treatment outcome in late-life depression. Int J Geriatr Psychiatry. 2019;34(5):730-737. doi:10.1002/gps.5079 30761621 PMC6480406

[6] Mulvahill JS, Nicol GE, Dixon D, . Effect of metabolic syndrome on late-life depression: associations with disease severity and treatment resistance. J Am Geriatr Soc. 2017;65(12):2651-2658. doi:10.1111/jgs.15129 29235659 PMC5730877

[7] Bingham KS, Whyte EM, Meyers BS, ; STOP-PD Study Group. Relationship between cerebrovascular risk, cognition, and treatment outcome in late-life psychotic depression. Am J Geriatr Psychiatry. 2015;23(12):1270-1275. doi:10.1016/j.jagp.2015.08.002 26560512 PMC4691567

[8] Barzilai N, Cuervo AM, Austad S. Aging as a biological target for prevention and therapy. JAMA. 2018;320(13):1321-1322. doi:10.1001/jama.2018.9562 30242337

[9] Kennedy BK, Berger SL, Brunet A, . Geroscience: linking aging to chronic disease. Cell. 2014;159(4):709-713. doi:10.1016/j.cell.2014.10.039 25417146 PMC4852871

[10] Brown PJ, Brennan N, Ciarleglio A, . Declining skeletal muscle mitochondrial function associated with increased risk of depression in later life. Am J Geriatr Psychiatry. 2019;27(9):963-971. doi:10.1016/j.jagp.2019.03.022 31104966 PMC7388241

[11] Diniz BS, Lin CW, Sibille E, . Circulating biosignatures of late-life depression (LLD): towards a comprehensive, data-driven approach to understanding LLD pathophysiology. J Psychiatr Res. 2016;82:1-7. doi:10.1016/j.jpsychires.2016.07.006 27447786 PMC9344393

[12] Diniz BS, Sibille E, Ding Y, . Plasma biosignature and brain pathology related to persistent cognitive impairment in late-life depression. Mol Psychiatry. 2015;20(5):594-601. doi:10.1038/mp.2014.76 25092249 PMC4494754

[13] Castro-Costa E, Diniz BS, Firmo JOA, . Diabetes, depressive symptoms, and mortality risk in old age: the role of inflammation. Depress Anxiety. 2019;36(10):941-949. doi:10.1002/da.22908 31066979

[14] de la Torre-Luque A, Ayuso-Mateos JL, Sanchez-Carro Y, de la Fuente J, Lopez-Garcia P. Inflammatory and metabolic disturbances are associated with more severe trajectories of late-life depression. Psychoneuroendocrinology. 2019;110:104443. doi:10.1016/j.psyneuen.2019.104443 31610452

[15] Diniz BS, Fisher-Hoch S, McCormick J. The association between insulin resistance, metabolic variables, and depressive symptoms in Mexican-American elderly: a population-based study. Int J Geriatr Psychiatry. 2018;33(2):e294-e299. doi:10.1002/gps.4792 28925048 PMC5773366

[16] López-Otín C, Blasco MA, Partridge L, Serrano M, Kroemer G. The hallmarks of aging. Cell. 2013;153(6):1194-1217. doi:10.1016/j.cell.2013.05.039 23746838 PMC3836174

[17] Schafer MJ, Zhang X, Kumar A, . The senescence-associated secretome as an indicator of age and medical risk. JCI Insight. 2020;5(12):133668. doi:10.1172/jci.insight.133668 32554926 PMC7406245

[18] Fafián-Labora JA, O’Loghlen A. Classical and nonclassical intercellular communication in senescence and ageing. Trends in Cell Biology. 2020;30(8):628-639. doi:10.1016/j.tcb.2020.05.003 32505550

[19] Murray GK, Lin T, Austin J, McGrath JJ, Hickie IB, Wray NR. Could polygenic risk scores be useful in psychiatry? a review. JAMA Psychiatry. 2021;78(2):210-219. doi:10.1001/jamapsychiatry.2020.3042 33052393

[20] Diniz BS, Vieira EM, Mendes-Silva AP, ; PACt‐MD Study Group. Mild cognitive impairment and major depressive disorder are associated with molecular senescence abnormalities in older adults. Alzheimers Dement (N Y). 2021;7(1):e12129. doi:10.1002/trc2.12129 33816758 PMC8012242

[21] Diniz BS, Reynolds III CF, Sibille E, Bot M, Penninx BWJH. Major depression and enhanced molecular senescence abnormalities in young and middle-aged adults. Transl Psychiatry. 2019;9(1):198. doi:10.1038/s41398-019-0541-3 31434875 PMC6704136

[22] Diniz BS, Reynolds CF III, Sibille E, . Enhanced molecular aging in late-life depression: the senescent-associated secretory phenotype. Am J Geriatr Psychiatry. 2017;25(1):64-72. doi:10.1016/j.jagp.2016.08.018 27856124 PMC5164865

[23] Mendes-Silva AP, Mwangi B, Aizenstein H, Reynolds CF III, Butters MA, Diniz BS. Molecular senescence is associated with white matter microstructural damage in late-life depression. Am J Geriatr Psychiatry. 2019;27(12):1414-1418. doi:10.1016/j.jagp.2019.06.006 31320246 PMC6842685

[24] Marshe VS, Maciukiewicz M, Hauschild A-C, . Genome-wide analysis suggests the importance of vascular processes and neuroinflammation in late-life antidepressant response. Transl Psychiatry. 2021;11(1):127. doi:10.1038/s41398-021-01248-3 33589590 PMC7884410

[25] Grzenda A, Siddarth P, Laird KT, Yeargin J, Lavretsky H. Transcriptomic signatures of treatment response to the combination of escitalopram and memantine or placebo in late-life depression. Mol Psychiatry. 2021;26(9):5171-5179. doi:10.1038/s41380-020-0752-2 32382137 PMC9922535

[26] Lenze EJ, Mulsant BH, Blumberger DM, . Efficacy, safety, and tolerability of augmentation pharmacotherapy with aripiprazole for treatment-resistant depression in late life: a randomised, double-blind, placebo-controlled trial. Lancet. 2015;386(10011):2404-2412. doi:10.1016/S0140-6736(15)00308-6 26423182 PMC4690746

[27] Kaneriya SH, Robbins-Welty GA, Smagula SF, . Predictors and moderators of remission with aripiprazole augmentation in treatment-resistant late-life depression: an analysis of the IRL-GRey randomized clinical trial. JAMA Psychiatry. 2016;73(4):329-336. doi:10.1001/jamapsychiatry.2015.3447 26963689 PMC4823175

[28] American Psychiatric Association. Diagnostic and Statistical Manual of Mental Disorders. 4th ed, text revision. American Psychiatric Association; 2000.

[29] Montgomery SA, Asberg M. A new depression scale designed to be sensitive to change. Br J Psychiatry. 1979;134:382-389. doi:10.1192/bjp.134.4.382 444788

[30] First M, Spitzer RL, Gibbon M, Williams JBW. Structured Clinical Interview for DSM-IV-TR Axis I Disorders, Research Version, Patient Edition. (SCID-I/P). Biometrics Research. New York State Psychiatric Institute; 2002.

[31] Miller MD, Paradis CF, Houck PR, . Rating chronic medical illness burden in geropsychiatric practice and research: application of the Cumulative Illness Rating Scale. Psychiatry Res. 1992;41(3):237-248. doi:10.1016/0165-1781(92)90005-N 1594710

[32] Homack S, Lee D, Riccio CA. Test review: Delis-Kaplan executive function system. J Clin Exp Neuropsychol. 2005;27(5):599-609. doi:10.1080/13803390490918444 16019636

[33] Lezak MD, Howieson DB, Bigler ED, Tranel D. Neuropsychological Assessment. 5th ed. Oxford University Press; 2012.

[34] Delis DC, Kaplan E, Kramer JH. Delis-Kaplan Executive Function System (D-KEFS) Examiner’s Manual. The Psychological Corporation; 2001.

[35] Coppé JP, Patil CK, Rodier F, . Senescence-associated secretory phenotypes reveal cell-nonautonomous functions of oncogenic RAS and the p53 tumor suppressor. PLoS Biol. 2008;6(12):2853-2868. doi:10.1371/journal.pbio.0060301 19053174 PMC2592359

[36] Gonzales MM, Krishnamurthy S, Garbarino V, . A geroscience motivated approach to treat Alzheimer’s disease: senolytics move to clinical trials. Mech Ageing Dev. 2021;200:111589. doi:10.1016/j.mad.2021.111589 34687726 PMC9059898

[37] Lin YF, Wang LY, Chen CS, Li CC, Hsiao YH. Cellular senescence as a driver of cognitive decline triggered by chronic unpredictable stress. Neurobiol Stress. 2021;15:100341. doi:10.1016/j.ynstr.2021.100341 34095365 PMC8163993

[38] Ferrucci L, Fabbri E. Inflammageing: chronic inflammation in ageing, cardiovascular disease, and frailty. Nat Rev Cardiol. 2018;15(9):505-522. doi:10.1038/s41569-018-0064-2 30065258 PMC6146930

[39] Eller T, Vasar V, Shlik J, Maron E. Pro-inflammatory cytokines and treatment response to escitalopram in major depressive disorder. Prog Neuropsychopharmacol Biol Psychiatry. 2008;32(2):445-450. doi:10.1016/j.pnpbp.2007.09.015 17976882

[40] Brunoni AR, Machado-Vieira R, Sampaio-Junior B, . Plasma levels of soluble TNF receptors 1 and 2 after tDCS and sertraline treatment in major depression: results from the SELECT-TDCS trial. J Affect Disord. 2015;185:209-213. doi:10.1016/j.jad.2015.07.006 26241865

[41] Bot M, Carney RM, Freedland KE, . Inflammation and treatment response to sertraline in patients with coronary heart disease and comorbid major depression. J Psychosom Res. 2011;71(1):13-17. doi:10.1016/j.jpsychores.2010.11.006 21665007 PMC3115530

[42] Carboni L, McCarthy DJ, Delafont B, . Biomarkers for response in major depression: comparing paroxetine and venlafaxine from two randomised placebo-controlled clinical studies. Transl Psychiatry. 2019;9(1):182. doi:10.1038/s41398-019-0521-7 31375659 PMC6677721

[43] Gasparini A, Callegari C, Lucca G, Bellini A, Caselli I, Ielmini M. Inflammatory biomarker and response to antidepressant in major depressive disorder: a systematic review and meta-analysis. Psychopharmacol Bull. 2022;52(1):36-52. 35342200 10.64719/pb.4425 PMC8896754

[44] Liu JJ, Wei YB, Strawbridge R, . Peripheral cytokine levels and response to antidepressant treatment in depression: a systematic review and meta-analysis. Mol Psychiatry. 2020;25(2):339-350. doi:10.1038/s41380-019-0474-5 31427752

[45] Ward J, Graham N, Strawbridge RJ, . Polygenic risk scores for major depressive disorder and neuroticism as predictors of antidepressant response: meta-analysis of three treatment cohorts. PLoS One. 2018;13(9):e0203896. doi:10.1371/journal.pone.0203896 30240446 PMC6150505

[46] Zheutlin AB, Ross DA. Polygenic risk scores: what are they good for? Biol Psychiatry. 2018;83(11):e51-e53. doi:10.1016/j.biopsych.2018.04.007 29759133 PMC6696910

[47] Zwicker A, Fabbri C, Rietschel M, . Genetic disposition to inflammation and response to antidepressants in major depressive disorder. J Psychiatr Res. 2018;105:17-22. doi:10.1016/j.jpsychires.2018.08.011 30130674

[48] Ridout KK, Ridout SJ, Price LH, Sen S, Tyrka AR. Depression and telomere length: a meta-analysis. J Affect Disord. 2016;191:237-247. doi:10.1016/j.jad.2015.11.052 26688493 PMC4760624

[49] Mendes-Silva AP, Vieira ELM, Xavier G, . Telomere shortening in late-life depression: a potential marker of depression severity. Brain Behav. 2021;11(8):e2255. doi:10.1002/brb3.2255 34152095 PMC8413729

[50] Wolkowitz OM, Mellon SH, Epel ES, . Resting leukocyte telomerase activity is elevated in major depression and predicts treatment response. Mol Psychiatry. 2012;17(2):164-172. doi:10.1038/mp.2010.133 21242992 PMC3130817

[51] Rasgon N, Lin KW, Lin J, Epel E, Blackburn E. Telomere length as a predictor of response to Pioglitazone in patients with unremitted depression: a preliminary study. Transl Psychiatry. 2016;6(1):e709. doi:10.1038/tp.2015.187 26731446 PMC5068869

[52] Pisanu C, Vitali E, Meloni A, . Investigating the role of leukocyte telomere length in treatment-resistant depression and in response to electroconvulsive therapy. J Pers Med. 2021;11(11):1100. doi:10.3390/jpm11111100 34834452 PMC8622097

[53] Rutherford BR, Taylor WD, Brown PJ, Sneed JR, Roose SP. Biological aging and the future of geriatric psychiatry. J Gerontol A Biol Sci Med Sci. 2017;72(3):343-352. doi:10.1093/gerona/glw241 27994004 PMC6433424

[54] He S, Sharpless NE. Senescence in health and disease. Cell. 2017;169(6):1000-1011. doi:10.1016/j.cell.2017.05.015 28575665 PMC5643029

[55] Baker DJ, Petersen RC. Cellular senescence in brain aging and neurodegenerative diseases: evidence and perspectives. J Clin Invest. 2018;128(4):1208-1216. doi:10.1172/JCI95145 29457783 PMC5873891

[56] Galluzzi L, Yamazaki T, Kroemer G. Linking cellular stress responses to systemic homeostasis. Nat Rev Mol Cell Biol. 2018;19(11):731-745. doi:10.1038/s41580-018-0068-0 30305710

[57] Varadhan R, Seplaki CL, Xue QL, Bandeen-Roche K, Fried LP. Stimulus-response paradigm for characterizing the loss of resilience in homeostatic regulation associated with frailty. Mech Ageing Dev. 2008;129(11):666-670. doi:10.1016/j.mad.2008.09.013 18938195 PMC2650618

[58] Katzir I, Adler M, Karin O, Mendelsohn-Cohen N, Mayo A, Alon U. Senescent cells and the incidence of age-related diseases. Aging Cell. 2021;20(3):e13314. doi:10.1111/acel.13314 33559235 PMC7963340

[59] Lee MB, Hill CM, Bitto A, Kaeberlein M. Antiaging diets: separating fact from fiction. Science. 2021;374(6570):eabe7365. doi:10.1126/science.abe7365 34793210 PMC8841109

[60] Englund DA, Sakamoto AE, Fritsche CM, . Exercise reduces circulating biomarkers of cellular senescence in humans. Aging Cell. 2021;20(7):e13415. doi:10.1111/acel.13415 34101960 PMC8282238

[61] Cabo Rd, Carmona-Gutierrez D, Bernier M, Hall MN, Madeo F. The search for antiaging interventions: from elixirs to fasting regimens. Cell. 2014;157(7). doi:10.1016/j.cell.2014.05.031 PMC4254402 24949965

[62] Uemura K, Makizako H, Lee S, . Behavioral protective factors of increased depressive symptoms in community-dwelling older adults: a prospective cohort study. Int J Geriatr Psychiatry. 2018;33(2):e234-e241. doi:10.1002/gps.4776 28841238

[63] Klil-Drori S, Klil-Drori AJ, Pira S, Rej S. Exercise intervention for late-life depression: a meta-analysis. J Clin Psychiatry. 2020;81(1):19r12877. doi:10.4088/JCP.19r12877 31967748

[64] Schuch FB, Vancampfort D, Rosenbaum S, . Exercise for depression in older adults: a meta-analysis of randomized controlled trials adjusting for publication bias. Braz J Psychiatry. 2016;38(3):247-254. doi:10.1590/1516-4446-2016-1915 27611903 PMC7194268

[65] Micco RD, Krizhanovsky V, Baker D, d’Adda di Fagagna F. Cellular senescence in ageing: from mechanisms to therapeutic opportunities. Nat Rev Mol Cell Bio. 2020;22(2):75-95. doi:10.1038/s41580-020-00314-w 33328614 PMC8344376

[66] Ogrodnik M, Zhu Y, Langhi LGP, . Obesity-induced cellular senescence drives anxiety and impairs neurogenesis. Cell Metab. 2019;29(5):1061-1077.e8. doi:10.1016/j.cmet.2018.12.008 30612898 PMC6509403

[67] Darrow SM, Verhoeven JE, Révész D, . The association between psychiatric disorders and telomere length: a meta-analysis involving 14,827 persons. Psychosom Med. 2016;78(7):776-787. doi:10.1097/PSY.0000000000000356 27359174 PMC5003712

[68] Diniz BS, Reynolds CF III, Butters MA, . The effect of gender, age, and symptom severity in late-life depression on the risk of all-cause mortality: the Bambuí Cohort Study of Aging. Depress Anxiety. 2014;31(9):787-795. doi:10.1002/da.22226 24353128 PMC4062606

[69] Naismith SL, Norrie LM, Mowszowski L, Hickie IB. The neurobiology of depression in later-life: clinical, neuropsychological, neuroimaging and pathophysiological features. Prog Neurobiol. 2012;98(1):99-143. doi:10.1016/j.pneurobio.2012.05.009 22609700

[70] Ly M, Karim HT, Becker JT, . Late-life depression and increased risk of dementia: a longitudinal cohort study. Transl Psychiatry. 2021;11(1):147. doi:10.1038/s41398-021-01269-y 33654078 PMC7925518

