# Supplementary material for: Association of Molecular Senescence Markers in Late-Life Depression With Clinical Characteristics and Treatment Outcome
Source: JAMA Netw Open. 2022 Jun 30;5(6):e2219678. doi: 10.1001/jamanetworkopen.2022.19678 (PMC9247739; doi:10.1001/jamanetworkopen.2022.19678)
Supplement: Supplement 1. — Trial Protocol [file jamanetwopen-e2219678-s001.pdf]

Incomplete Response in Late Life Depression: Getting to Remission (IRL GREY) (IRL GREY) - Protocol

Study Description

Brief Summary:

The primary aims of this study are to:

1. Assess the efficacy of aripiprazole augmentation for the acute and continuation treatment of TRLLD.

Hypothesis 1: Patients with TRLLD (defined as those who do not remit after 12 weeks of acute treatment withvenlafaxine XR) will have a higher rate of remission with aripiprazole than with placebo augmentation (primary outcome) and greater improvement in depressive symptoms and stability of remission (secondary outcomes).

2. Assess the tolerability of aripiprazole in TRLLD with a focus on adiposity and akathisia/restlessness.

Hypothesis 2: Aripiprazole will be associated with a higher rate of clinically significant akathisia and increasedadiposity than placebo.

The Secondary/exploratory aims of this study are to:

1. Examine anxiety, medical burden, and executive impairment as moderators of aripiprazole augmentationefficacy in TRLLD.

Hypothesis 3: Pre-levels of anxiety symptoms, medical burden, and executive impairment will be treatment-specific factors: they will moderate the efficacy of aripiprazole augmentation. The aripiprazole-placebo difference will be greater in individuals with these variables, compared to those without these variables because these three factors will be associated with a decreased likelihood that "staying the course" with venlafaxine monotherapy will achieve remission.

2. Examine genetic predictors (phase 1) and moderators (phase 2-3) of treatment outcomes, while controllingfor drug exposure.

Hypothesis 4: Selected polymorphisms will reduce remission rate with venlafaxine and will reduce efficacy andtolerability with aripiprazole.

Detailed Description:

Incomplete response in the treatment of late-life depression (LLD) is a large public health challenge: at least 50% ofolder people fail to respond adequately to antidepressant pharmacotherapy, even under optimal treatment conditions. Treatment resistant late-life depression (TRLLD) increases risk for early relapse, undermines adherenceto treatment for coexisting medical disorders, amplifies disability and cognitive impairment, imposes greater burden on family caregivers, and increases the risk for early mortality, including suicide. Getting to and sustaining remissionis the primary goal of treatment, yet there is a paucity of controlled studies of how best to manage TRLLD.

This is a multi-site study being conducted by 3 sites: University of Pittsburgh, University of Toronto, and WashingtonUniversity. We propose to enroll 500 subjects aged 60 and older with major depressive disorder at this site and treatthem openly for 12 weeks with venlafaxine XR (up to 300mg/d) (phase 1). Participants meeting criteria for incomplete response will be randomly assigned to receive either aripiprazole (2-15 mg/d; target dose: 10 mg/d) or placebo augmentation (adding a pill without active medicine) of venlafaxine for 12 weeks (phase 2), with the goal of achieving remission (MADRS≤10 for two consecutive assessments). Those who remit in phase 2 will receive continuation treatment, with the same double-blinded intervention to which they were randomly assigned (phase 3), for 12 weeks to determine the stability of remission. Efficacy and tolerability data will provide a clinically informative estimate of benefits and risks of aripiprazole augmentation for TRLLD.

In addition to the primary goal of assessing these benefits and risks, we will develop evidence relevant to personalized treatment for LLD by testing the roles of clinical (comorbid anxiety, medical burden, and executive impairment) and genetic (selected polymorphisms in serotonin, norepinephrine, and dopamine genes) variables, while controlling for variability in drug exposure for efficacy and tolerability analyses. This approach will allow us todistinguish treatment-specific resistance factors versus general prognostic factors.

Study design

Study Type: Interventional (Clinical trial)
Actual Enrollment: 468 participants
Allocation: Randomized Intervention Model: Parallel Assignment Masking: Triple (Participant, Investigator, Outcomes Assessor)
Primary Purpose: Treatment
Start Date: August 2009
Completion Date: September 2014

Arms and interventions

Arm	Intervention/treatment
Experimental: 1: venlafaxine plus aripiprazole<br>antidepressant (venlafaxine) plus aripiprazol or venlafaxine plus placebo	Drug: venlafaxine XR plus aripiprazole<br>Dosage varies. Subject remains on antidepressant throughout the 36 week study. Will be randomized toaripiprazole or placebo for up to 24 weeks.<br>Other Name: effexor XR, abilify
Experimental: 2: Placebo Comparator<br>antidepressant (venlafaxine) plus aripiprazol or venlafaxine plus placebo	Drug: venlafaxine plus placebo<br>Dosage varies. Subject remains on antidepressant throughout the 36 week study. Will be randomized toaripiprazole or placebo for up to 24 weeks.

Outcome measures

Primary Outcome Measures :

1. Percentage of Subjects Who Met Criteria for Remission Based on the Montgomery-Asberg Depression RatingScale (MADRS) [Time Frame: 12 weeks]

   The Montgomery-Asberg Depression Rating Scale (MADRS) is a clinician rated ten item instrument assessing depression symptoms. Possible scores range from 0-60; higher scores indicate greater severity ofdepression. Remission defined as score of 10 or less based on the MADRS.

2. Akathisia [Time Frame: 12 weeks]

   Percentage of participants who developed clinically significant akathisia.

3. Weight [Time Frame: Baseline through12 weeks]Weight change in kilograms

4. Parkinsonism [Time Frame: 12weeks]

   Percentage of participants who develop signs of parkinsonism

Secondary Outcome Measures :

1. Emergent Suicidal Ideation in Those With no Ideation at the Start of Treatment [Time Frame: 12 weeks]percentage of participants who reported suicidal ideation during treatment but not at baseline

QTc Prolongation on EKG (to Greater or Equal to 480 Msec) [Time Frame: 12 weeks

Eligibility Criteria

Ages Eligible for Study: 60 Years and older (Adult, Older Adult)Sexes Eligible for Study: All

Accepts Healthy Volunteers: No

Criteria

Inclusion Criteria:

1. Age > 60 years.
2. Major depressive disorder (MDD), single or recurrent, as diagnosed by the SCID-IV.
3. MADRS ≥ 15

Exclusion criteria

1. Inability to provide informed consent.
2. Depressive symptoms not severe enough (i.e., MADRS < 15) at the baseline assessments.
3. Dementia based upon DSM-IV criteria as well as a Folstein MMSE score of less than 24. Patients screened out due to dementia will be referred to a memory clinic or to the UPMC Alzheimer's Disease Research Centerfor evaluation to clarify the presence or absence of a dementia.
4. Lifetime diagnosis of bipolar I or II disorder, schizophrenia, schizoaffective disorder, schizophreniform disorder, delusional disorder, or current psychotic symptoms, as diagnosed by the SCID. A recommendationfor psychiatric referral will be made in these cases.
5. Abuse of or dependence on alcohol or other substances within the past 3 months as determined by SCID,and confirmed by study physician interview.
6. High risk for suicide (e.g., active SI and/or current/recent intent or plan) AND unable to be managed safely inthe clinical trial (e.g., unwilling to be hospitalized). Urgent psychiatric referral will be made in these cases.
7. Contraindication to venlafaxine XR or aripiprazole as determined by study physician including history of intolerance of either venlafaxine XR or aripiprazole in the study target dosage range (venlafaxine XR at

up to225 mg/day; aripiprazole at up to 15mg/day).

8. Failure to respond to at least 6 weeks of venlafaxine (>225 mg/d) plus aripiprazole (>10 mg/d).
9. Inability to communicate in English (i.e., interview cannot be conducted without an interpreter; subject largelyunable to understand questions and cannot respond in English).
10. Non-correctable clinically significant sensory impairment (i.e., cannot hear well enough to cooperate withinterview)
11. Unstable medical illness, including delirium, uncontrolled diabetes mellitus, hypertension, hyperlipidemia, or cerebrovascular or cardiovascular risk factors that are not under medical management. This will be determined based on information from the patient's personal physician's and study physician clinical judgment. Referral to the patient's personal physician or to a general practitioner will be made in these cases.
12. Subjects taking psychotropic medications that cannot be safely tapered or discontinued prior to study initiation: this would include patients on Monoamine Oxidase Inhibitors (MAOI) who would need to be off the MAOI for 14 days to be eligible for the study to avoid adverse drug interactions. Patients will not be allowed to take antidepressant or atypical antipsychotic medication other than the study medication, unless it is a lowdose antidepressant prescribed for chronic pain that would not be medically advisable to stop (e.g., amitryptyline 50mg). If a patient's depression is adequately treated on his/her psychotropic medication, he/she would not be eligible for the study. If a patient failed a trial of venlafaxine (12 weeks of treatment with venlafaxine including at least 6 weeks on 300mg/day), he/she would not be eligible. The following are allowed: benzodiazepines up to 2mg/d lorazepam equivalent; other sedative-hypnotics (e.g., zolpidem, zaleplon, eszopiclone); gabapentin if prescribed for non-psychiatric indication (e.g., neuropathy). Except for MAOIs, there is really no clinical rationale to exclude patients on specific concomitant medications unless they are medically unstable (in which case they are excluded from participation). As noted, patients on an MAOI would need to be off the MAOI for 14 days to protect from adverse drug interactions.

Additional information

Publications of Results:

Lenze EJ, Mulsant BH, Blumberger DM, Karp JF, Newcomer JW, Anderson SJ, Dew MA, Butters MA, Stack JA, Begley AE, Reynolds CF 3rd. Efficacy, safety, and tolerability of augmentation pharmacotherapy with aripiprazolefor treatment-resistant depression in late life: a randomised, double-blind, placebo-controlled trial. Lancet. 2015 Dec 12;386(10011):2404-12. doi: 10.1016/S0140-6736(15)00308-6. Epub 2015 Sep 27. Erratum in: Lancet. 2015Dec 12;386(10011):2394.

Publications automatically indexed to this study by ClinicalTrials.gov Identifier (NCT Number):

Altmann H, Stahl ST, Gebara MA, Lenze EJ, Mulsant BH, Blumberger DM, Reynolds CF 3rd, Karp JF. Coprescribed Benzodiazepines in Older Adults Receiving Antidepressants for Anxiety and Depressive Disorders:Association With Treatment Outcomes. J Clin Psychiatry. 2020 Sep 29;81(6). pii: 20m13283. doi: 10.4088/JCP.20m13283.

Buchalter ELF, Oughli HA, Lenze EJ, Dixon D, Miller JP, Blumberger DM, Karp JF, Reynolds CF 3rd, Mulsant BH.Predicting Remission in Late-Life Major Depression: A Clinical Algorithm Based Upon Past Treatment History. J Clin Psychiatry. 2019 Dec 10;80(6). pii: 18m12483. doi: 10.4088/JCP.18m12483.

Wei W, Karim HT, Lin C, Mizuno A, Andreescu C, Karp JF, Reynolds CF 3rd, Aizenstein HJ. Trajectories in Cerebral Blood Flow Following Antidepressant Treatment in Late-Life Depression: Support for the Vascular Depression Hypothesis. J Clin Psychiatry. 2018 Oct 23;79(6). pii: 18m12106. doi: 10.4088/JCP.18m12106.

Hsu JH, Mulsant BH, Lenze EJ, Sanches M, Karp JF, Reynolds CF, Blumberger DM. Clinical Predictors of Extrapyramidal Symptoms Associated With Aripiprazole Augmentation for the Treatment of Late-Life Depression ina Randomized Controlled Trial. J Clin Psychiatry. 2018 Jun 19;79(4). pii: 17m11764. doi: 10.4088/JCP.17m11764.

Cristancho P, Lenze EJ, Dixon D, Miller JP, Mulsant BH, Reynolds CF 3rd, Butters MA. Executive Function PredictsAntidepressant Treatment Noncompletion in Late-Life Depression. J Clin Psychiatry. 2018 May/Jun;79(3). pii: 16m11371. doi: 10.4088/JCP.16m11371.

Marshe VS, Maciukiewicz M, Rej S, Tiwari AK, Sibille E, Blumberger DM, Karp JF, Lenze EJ, Reynolds CF 3rd, Kennedy JL, Mulsant BH, Müller DJ. Norepinephrine Transporter Gene Variants and Remission From DepressionWith Venlafaxine Treatment in Older Adults. Am J Psychiatry. 2017 May 1;174(5):468-475. doi: 10.1176/appi.ajp.2016.16050617. Epub 2017 Jan 10.

Smagula SF, Karim HT, Lenze EJ, Butters MA, Wu GF, Mulsant BH, Reynolds CF, Aizenstein HJ. Gray matter regions statistically mediating the cross-sectional association of eotaxin and set-shifting among older adults with major depressive disorder. Int J Geriatr Psychiatry. 2017 Dec;32(12):1226-1232. doi: 10.1002/gps.4585. Epub 2016Sep 19.

Smagula SF, Lotrich FE, Aizenstein HJ, Diniz BS, Krystek J, Wu GF, Mulsant BH, Butters MA, Reynolds CF 3rd, Lenze EJ. Immunological biomarkers associated with brain structure and executive function in late-life depression: exploratory pilot study. Int J Geriatr Psychiatry. 2017 Jun;32(6):692-699. doi: 10.1002/gps.4512. Epub 2016 Jun 10.

Kaneriya SH, Robbins-Welty GA, Smagula SF, Karp JF, Butters MA, Lenze EJ, Mulsant BH,

Blumberger D, Anderson SJ, Dew MA, Lotrich F, Aizenstein HJ, Diniz BS, Reynolds CF 3rd. Predictors and Moderators of Remission With Aripiprazole Augmentation in Treatment-Resistant Late-Life Depression: An Analysis of the IRL-GRey Randomized Clinical Trial. JAMA Psychiatry. 2016 Apr;73(4):329-36. doi: 10.1001/jamapsychiatry.2015.3447.

Kasckow J, Youk A, Anderson SJ, Dew MA, Butters MA, Marron MM, Begley AE, Szanto K, Dombrovski AY, Mulsant BH, Lenze EJ, Reynolds CF 3rd. Trajectories of suicidal ideation in depressed older adults undergoing antidepressant treatment. J Psychiatr Res. 2016 Feb;73:96-101. doi: 10.1016/j.jpsychires.2015.11.004. Epub 2015Nov 19.

Smagula SF, Butters MA, Anderson SJ, Lenze EJ, Dew MA, Mulsant BH, Lotrich FE, Aizenstein H, Reynolds CF 3rd. Antidepressant Response Trajectories and Associated Clinical Prognostic Factors Among Older Adults. JAMAPsychiatry. 2015 Oct;72(10):1021-8. doi: 10.1001/jamapsychiatry.2015.1324.

Hall CA, Simon KM, Lenze EJ, Dew MA, Begley A, Butters MA, Blumberger DM, Stack JA, Mulsant B, Reynolds CF 3rd. Depression Remission Rates Among Older Black and White Adults: Analyses From the IRL-GREY Trial. Psychiatr Serv. 2015 Dec 1;66(12):1303-11. doi: 10.1176/appi.ps.201400480. Epub 2015 Aug 17.

Joel I, Begley AE, Mulsant BH, Lenze EJ, Mazumdar S, Dew MA, Blumberger D, Butters M, Reynolds CF 3rd; IRLGREY Investigative Team. Dynamic prediction of treatment response in late-life depression. Am J Geriatr Psychiatry. 2014 Feb;22(2):167-76. doi: 10.1016/j.jagp.2012.07.002. Epub 2013 Feb 6.
