# Supplementary material for: Association of Molecular Senescence Markers in Late-Life Depression With Clinical Characteristics and Treatment Outcome
Source: JAMA Netw Open. 2022 Jun 30;5(6):e2219678. doi: 10.1001/jamanetworkopen.2022.19678 (PMC9247739; doi:10.1001/jamanetworkopen.2022.19678)
Supplement: Supplement 2. — eTable 1. Comparison Between Individual SASP Factors Between Nonremission vs Remission Groups eTable 2. Logistic Regression of Each Individual SASP Factor to Predict Treatment Outcome [file jamanetwopen-e2219678-s002.pdf]

Supplemental Online Content

Diniz BS, Mulsant BH, Reynolds CF III, et al. Association of molecular senescence markers in late-life depression with clinical characteristics and treatment outcome. *JAMA Netw Open*. 2022;5(6):e2219678. doi:10.1001/jamanetworkopen.2022.19678

eTable 1. Comparison Between Individual SASP Factors Between Nonremission vs Remission Groups

eTable 2. Logistic Regression of Each Individual SASP Factor to Predict Treatment Outcome

This supplemental material has been provided by the authors to give readers additional information about their work.

© 2022 Diniz BS et al. *JAMA Network Open.*

eTable 1. Comparison Between Individual SASP Factors Between Nonremission vs. Remission Groups

	Unadjusted										Adjusted*		
	Whole sample			Treatment outcome									
				Remission		Treatment resistance							
	SASP index weight	Mean	SD	Mean	SD	Mean	SD	t	df	Sig.	F	df	sig
IL-6	0.364	2.03	0.44	1.98	0.48	2.07	0.41	-1.98	414	0.05			
gp130	0.403	16.47	0.70	16.48	0.40	16.47	0.85	0.03	414	0.97			
IL-8	0.47	2.96	0.73	2.90	0.74	3.00	0.72	-1.29	414	0.20			
uPAR	0.583	9.81	0.58	9.82	0.54	9.81	0.61	0.27	414	0.79			
MIF	0.304	10.99	1.10	11.00	1.23	10.97	1.01	0.28	414	0.78			
MCP-1	0.35	8.36	0.71	8.30	0.68	8.40	0.73	-1.52	414	0.13			
Osteoprotegerin	0.565	9.96	0.45	9.95	0.46	9.97	0.45	-0.47	414	0.64			
IL1b	0.536	3.04	0.45	3.00	0.50	3.08	0.41	-1.78	414	0.08			
**MIP-3α**	**0.481**	**4.18**	**1.90**	**3.94**	**1.98**	**4.36**	**1.83**	**-2.22**	**414**	**0.03**	0.93	6,407	0.33
MIP-1α	0.572	5.43	1.48	5.41	1.53	5.44	1.45	-0.22	414	0.83			
MIP-1β	0.556	8.99	0.23	8.98	0.27	8.99	0.20	-0.51	414	0.61			
MCP-4	0.347	7.29	0.67	7.25	0.69	7.32	0.66	-1.09	414	0.27			
GMCSF	0.466	0.14	1.04	0.07	1.15	0.19	0.95	-1.12	414	0.26			
ICAM-1	0.416	18.20	0.85	18.21	0.94	18.19	0.77	0.27	414	0.78			
**TNFRII**	**0.612**	**11.38**	**0.60**	**11.30**	**0.62**	**11.43**	**0.59**	**-2.21**	**414**	**0.03**	0.29	6,407	0.59
TNFRI	0.719	10.65	0.69	10.58	0.72	10.70	0.65	-1.75	414	0.08			
PLGF	0.211	2.15	1.57	2.05	1.65	2.23	1.50	-1.13	414	0.26			
GRO-α	0.169	5.80	1.89	5.60	1.84	5.94	1.93	-1.80	414	0.07			
IGFBP-2	-0.104	15.45	2.41	15.54	2.41	15.38	2.41	0.65	414	0.52			
TIMP-1	-0.219	12.40	2.45	12.58	2.46	12.26	2.45	1.28	414	0.20			
IGFBP-6	-0.185	17.38	0.83	17.32	0.73	17.42	0.89	-1.27	414	0.20			

© 2022 Diniz BS et al. *JAMA Network Open.*

Angiogenin	-0.364	18.01	0.91	17.98	0.91	18.03	0.90	-0.58	414	0.57			

All biomarkers data is log_2 transformed.

*Adjusted by self-reported male sex, MADRS scores, duration of depressive episode, diastolic blood pressure levels.

© 2022 Diniz BS et al. *JAMA Network Open.*

eTable 2. Logistic Regression of Each Individual SASP Factor to Predict Treatment Outcome

	Unadjusted				Adjusted*			
		$CI_{95\%}$				$CI_{95\%}$		
	OR	Lower	Upper	p-value	OR	Lower	Upper	p-value
IL6	1.39	0.79	2.44	0.25	-	-	-	-
gp130	1.02	0.70	1.48	0.94	-	-	-	-
IL 8	1.12	0.82	1.52	0.48	-	-	-	-
uPAR	0.88	0.56	1.36	0.56	-	-	-	-
MIF	0.94	0.78	1.14	0.54	-	-	-	-
MCP1	1.21	0.89	1.67	0.23	-	-	-	-
Osteoprotegerin	0.79	0.46	1.34	0.37	-	-	-	-
IL1b	1.52	0.78	2.94	0.22	-	-	-	-
MIP3a	1.08	0.96	1.22	0.18	-	-	-	-
MIP1a	1.03	0.90	1.19	0.72	-	-	-	-
MIP1b	0.44	0.12	1.60	0.22	-	-	-	-
MCP4	1.02	0.72	1.43	0.93	-	-	-	-
GMCSF	1.09	0.88	1.35	0.44	-	-	-	-
ICAM1	0.93	0.73	1.19	0.56	-	-	-	-
TNFRII	1.30	0.82	2.08	0.27	-	-	-	-
TNFRI	1.03	0.72	1.49	0.85	-	-	-	-
GROa	1.08	0.89	1.31	0.43	-	-	-	-
PLGF	1.08	0.96	1.23	**0.21**				
**IGFBP2**	1.28	1.01	1.63	**0.04**	1.00	0.92	1.09	0.95
TIMP1	0.84	0.66	1.05	0.13	-	-	-	-
IGFBP6	1.01	0.63	1.63	0.96	-	-	-	-
Angiogenin	1.06	0.70	1.63	0.78	-	-	-	-

* Adjusted by self-reported male sex, MADRS scores, and scores on cognitive inhibition and set-shifting tasks.

© 2022 Diniz BS et al. *JAMA Network Open.*
